# Supplementary material for: Altered Resting-State Brain Activity and Functional Connectivity in Post-Stroke Apathy: An fMRI Study
Source: Brain Sci. 2023 Apr 27;13(5):730. doi: 10.3390/brainsci13050730 (PMC10216119; doi:10.3390/brainsci13050730)
Supplement: Supplementary file 1 [file brainsci-13-00730-s001.zip › brainsci-2300325-supplementary.pdf]

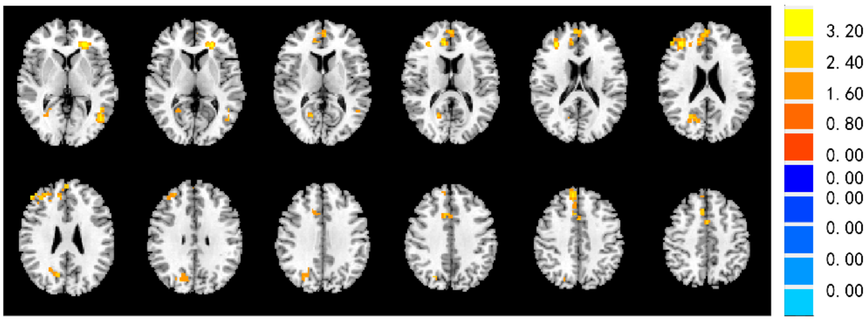

Figure S1. One-way ANOVA of PSA, nPSA and HCs. Voxel-level P < 0.05, cluster-level P<0.05, GRF correction. The yellow areas represent the regions which have altered fALFF.

PSA: postpost-stroke apathy; nPSA: nonpost-stroke apathy; HCs: healthy controls; fALFF: fractional amplitude of low frequency fluctuation; GRF correction: Gaussian random-field correction.
